# Virulence Potential and Antimicrobial Resistance of *Listeria monocytogenes* Isolates Obtained from Beef and Beef-Based Products Deciphered Using Whole-Genome Sequencing

**DOI:** 10.3390/microorganisms12061166

**Published:** 2024-06-08

**Authors:** Ayanda Manqele, Abiodun Adesiyun, Thendo Mafuna, Rian Pierneef, Rebone Moerane, Nomakorinte Gcebe

**Affiliations:** 1 Department of Production Animal Studies, Faculty of Veterinary Science, University of Pretoria, Pretoria 0110, South Africa; 2 Agricultural Research Council-Onderstepoort Veterinary Research, Pretoria 0110, South Africa; 3 Department of Basic Veterinary Sciences, University of the West Indies, St. Augustine 999183, Trinidad and Tobago; 4 Department of Biochemistry, University of Johannesburg, Johannesburg 20062028, South Africa; 5 Department of Biochemistry, Genetics and Microbiology, University of Pretoria, Pretoria 0001, South Africa; 6 Centre for Bioinformatics and Computational Biology, University of Pretoria, Pretoria 0001, South Africa; 7 SARChI Chair: Marine Microbiomics, microbiome@UP, Department of Biochemistry, Genetics and Microbiology, University of Pretoria, Pretoria 0001, South Africa

**Keywords:** *Listeria monocytogenes*, beef and beef-based products, whole-genome sequencing, type VII secretion system

## Abstract

*Listeria monocytogenes* is a ubiquitous bacterial pathogen that threatens the food chain and human health. In this study, whole-genome sequencing (WGS) was used for the genomic characterization of *L. monocytogenes* (n = 24) from beef and beef-based products. Multilocus Sequence Type (MLST) analysis revealed that ST204 of CC204 was the most common sequence type (ST). Other sequence types detected included ST1 and ST876 of CC1, ST5 of CC5, ST9 of CC9, ST88 of CC88, ST2 and ST1430 of CC2, and ST321 of CC321. Genes encoding for virulence factors included complete LIPI-1 (*pfrA*-*hly*-*plcA*-*plcB*-*mpl*-*actA*) from 54% (13/24) of the isolates of ST204, ST321, ST1430, and ST9 and internalin genes *inlABC* that were present in all the STs. All the *L. monocytogenes* STs carried four intrinsic/natural resistance genes, *fosX*, *lin*, *norB*, and *mprF*, conferring resistance to fosfomycin, lincosamide, quinolones, and cationic peptides, respectively. Plasmids pLGUG1 and J1776 were the most detected (54% each), followed by pLI100 (13%) and pLM5578 (7%). The prophage profile, vB_LmoS_188, was overrepresented amongst the isolates, followed by LP_101, LmoS_293_028989, LP_030_2_021539, A006, and LP_HM00113468. *Listeria* genomic island 2 (LGI-2) was found to be present in all the isolates, while *Listeria* genomic island 3 (LGI-3) was present in a subset of isolates (25%). The type VII secretion system was found in 42% of the isolates, and sortase A was present in all *L. monocytogenes* genomes. Mobile genetic elements and genomic islands did not harbor any virulence, resistance, or environmental adaptation genes that may benefit *L. monocytogenes*. All the STs did not carry genes that confer resistance to first-line antibiotics used for the treatment of listeriosis. The characterization of *L. monocytogenes* in our study highlighted the environmental resistance and virulence potential of *L. monocytogenes* and the risk posed to the public, as this bacterium is frequently found in food and food processing environments.

## 1. Introduction

*Listeria monocytogenes* are Gram-positive bacteria that are abundantly distributed in the environment and frequently associated with food processing environments and food products, especially ready-to-eat (RTE) products [1,2]. The occurrence of *L. monocytogenes* in food processing environments is often supported by its ability to tolerate adverse environmental conditions such as a wide range of pH (4.1–9.6), high salt levels (10% NaCl), adverse temperature conditions (0–45 °C), low water activity (0.92), and ability to form biofilms [3]. Additionally, the resistance of *L. monocytogenes* to disinfecting agents promotes its colonization of the food processing environment. The disinfectants commonly used are quaternary ammonium chloride (QAC) products, such as benzalkonium chloride (BC) [4]. *L. monocytogenes* has acquired resistance to BC via efflux pumps encoded by the *bcrABC* cassette, the *emrE* gene, plasmid-borne *emrC*, and *qahH* [5,6]. The resistance is also exacerbated by using cleaning agents without adhering to manufacturers’ instructions and over-exposure of *L. monocytogenes* to disinfectants [7,8]. It, therefore, becomes relatively difficult to control, prevent, and manage colonization, persistence, and infection due to *L. monocytogenes* [9].

Transmission of *Listeria* to humans is mainly via consuming contaminated food, and the risk of infection is higher in immunocompromised individuals [10]. In immunocompromised individuals, infection with *L. monocytogenes* can be invasive and develop into severe diseases that require antimicrobial therapy [11]. Antimicrobial treatment for listeriosis includes using beta-lactam antibiotics such as amoxicillin, penicillin or ampicillin, and gentamycin. Trimethoprim-sulfamethoxazole is given to patients with allergic reactions to penicillin, whereas physicians resort to using tetracycline and erythromycin for patients not responding accordingly to first-line antibiotics [12]. Even though *Listeria* is generally susceptible to a broad panel of antimicrobial agents that act against Gram-positive bacteria, intrinsic resistance to the antibiotics fosfomycin and cephalosporins has been documented [13]. Over the years, numerous studies have reported the antibiotic resistance of *L. monocytogenes* strains originating from food and food environments [14,15]. Varying factors, such as the application of antimicrobials in food animals for growth, disease prevention, and treatment of diseases, affect and influence antimicrobial resistance patterns in bacteria [16,17]. Acquired resistance in *L. monocytogenes* is attributed to mobile genetic elements, transposons, and plasmids [18]. The transfer of mobile genetic elements from enterococci and streptococci has contributed immensely to the development of antimicrobial resistance in *L. monocytogenes* [19]. Therefore, efficient monitoring of the dissemination and emergence of *L. monocytogenes*-resistant strains is paramount for prescribing effective antimicrobial therapy [14].

*Listeria monocytogenes* pathogenicity depends on its ability to bypass host barriers, escape the host immune system, invade and survive in the host cell, and tamper with the host’s cellular mechanisms [20]. These stages of infection are mediated by virulence genes that are harbored within the core genome and accessory genomes in some *L. monocytogenes* strains [21]. Pathogenicity Islands (PIs) comprise sets of virulence genes; these islands play an indispensable role in establishing infection and functional diversity amongst the *Listeria* isolates [14]. Four pathogenicity islands have been identified in *L. monocytogenes* [22]. *Listeria* pathogenicity island 1 (LIPI-1) consisting of a set of six genes (*hly*, *mpl*, *actA*, *plcA*, *plcB*, and *prfA*), is significant for virulence as all the genes play varying important roles in *Listeria* pathogenesis [14]. The internalins are other important virulence proteins that are essential for *Listeria* pathogenesis, and 11 internalin genes (*inlA*, *inlB*, *inlC*, *inlC2*, *inlD*, *inlE*, *inlF*, *inlG*, *inlH*, *inlI*, and *inlJ*) have been identified in *Listeria* [23]. Internalins are important for adhesion and host invasion of non-phagocytic cells [24,25].

Another important virulence component in *L. monocytogenes* is the type VII secretion system (T7SS) [26]. The T7SS is a protein secretion system used by *L. monocytogenes* for the secretion of proteins, including essential virulence proteins [27]. The secretion systems that make up the T7SS include the Sec system, the Tat (Twin-arginine translocation) pathway, the FEA (flagella export apparatus), the FPE (frimbilin-protein exporter), the Holins, and the Wss (WXG100 secretion system), which are proteins with WXG-motif of ~100 amino acids, and these systems are responsible for translocation of secreted proteins across the cytoplasmic membrane [28,29]. Amongst these secretion systems, the Sec system is regarded as the major secretion system for most proteins. It is responsible for the secretion of the majority of virulence proteins [30,31]. The Sec system incorporates the SecA component important for survival and viability; SecA contains two homologs known as SecA1 and SecA2 [30,31]. SecA2 is necessary for pathogenesis as it is involved in the secretion of proteins that are also involved in virulence [31,32]. SecA1 is also essential for the secretion of proteins, but not virulent proteins [31]. The mutation of SecA2 has been demonstrated to result in reduced virulence in *L. monocytogenes* [33,34]. Proteins such as Iap (invasion-associated protein), NamA (N-acetylmuramidase), and FbpA (fibronectin-binding protein) are reportedly dependent on SecA2 for secretion [28].

Whole-genome sequencing (WGS) has been applied to molecularly characterize *L. monocytogenes* isolates from food sources [35,36]. Similarly, in South Africa, WGS has been applied in the characterization of *L. monocytogenes* isolates from various meat products, including RTE products from different animal species in South Africa [37,38,39,40,41]. WGS was also used to resolve the large outbreak of listeriosis in South Africa during 2017/2018 [41]. With all the data generated through WGS in South Africa, data regarding the genomic characteristics of *L. monocytogenes* from beef and beef-based products is still lacking. Furthermore, studies have focused on the characterization of virulence genes in *L. monocytogenes* and the role of virulence proteins in *L. monocytogenes*. However, no studies have investigated the full virulence potential by screening the isolates for the presence of the T7SS, which is important for the secretion of the virulence proteins. Therefore, this study aimed to apply WGS and bioinformatics tools for molecular characterization of *L. monocytogenes* and assessing the complete virulence potential of this pathogen.

## 2. Materials and Methods

### 2.1. Origin of Isolates

*L. monocytogenes* (n = 24) isolates that originated from beef and beef-based products that were purchased from retailers in Mpumalanga and North-West provinces were used in this study. Beef products were defined as beef meat cuts from cattle, and beef-based products were defined as meat products derived from beef meat that have been processed before being sold to consumers. These isolates had been previously preserved in 70% glycerol and kept at −20 °C. To resuscitate the isolates, they were thawed in room temperature and vortexed for 30 s. A volume of 0.1 mL was inoculated into 9 mL brain heart infusion (BHI) broth (Oxoid, Basingstoke, UK) and incubated at 37 °C for ±24 h. Post-incubation, the broth was inoculated onto Brilliance *Listeria* agar (BLA) (Oxoid, Basingstoke, UK) and incubated at 37 °C for 48 h for re-subculturing. The pure culture was then subjected to genomic DNA extraction.

### 2.2. Genomic DNA Extraction

The genomic DNA was extracted using a High Pure PCR Template Preparation kit (Roche, Mannheim, Germany), according to the manufacturer’s instructions. Cultures of *L. monocytogenes* prepared from a single colony on a Brilliance *Listeria* Agar plate were used for DNA extraction. A loop full of *L. monocytogenes* culture was suspended in 200 µL distilled water, followed by adding 200 µL of Binding Buffer and 40 µL Proteinase K mix immediately; the mixture was incubated for 10 min at 70 °C using a heating block. Post-incubation, 100 µL of isopropanol was added, and the mixture was applied to a High Pure Filter tube and centrifuged at 8000× *g* for 1 min. The flow-through and collection tubes were discarded. Then, 500 µL of inhibitor removal buffer was added into the High Pure filter tube and centrifuged at 8000× *g* for 1 min. The flow-through and collection tubes were discarded, and 500 µL of wash buffer was added to the filter tube and centrifuged at 8000× *g* for 1 min. This washing step was repeated twice, and the filter tube was centrifuged at 13,000× *g* for 10 s. The flow-through and collection tubes were discarded. Then, 200 µL of Elution buffer (preheated at 70 °C) and new collection tubes were added, and the mixture was centrifuged at 8000× *g* for 1 min. The extracted DNA was stored at −20 °C before being submitted for whole-genome sequencing.

### 2.3. Whole-Genome Sequencing, Quality Control (Trimming), De Novo Assembly, and Genome Annotation

Whole-genome sequencing of the extracted genomic DNA was performed at the Agricultural Research Council-Onderstepoort Veterinary Research-Biotechnology Platform, South Africa. The DNA Libraries were prepared using Nextera XT DNA library preparation kit (Illumina, San Diego, CA, USA), followed by a 2 × 300 paired-end sequencing runs with 100× coverage using Illumina MiSeq equipment (Illumina, San Diego, CA, USA) [42,43]. Raw reads were subjected to the removal of adaptors and barcode sequences that were added during library generation. Trimming also removed low- or poor-quality reads. Trimmomatic version 0.39 was also used to trim and remove sequencing. The quality checks were performed using FastQC v.0.11.9 and the reports regarding basic statistics and data on the reads were also generated using FastQC v.011.9 [44]. Trimmed reads were assembled using SPAdes software (v 3.13) into draft genomes [45]. Quast software v5.0.2 assessed assembly quality [45]. All de novo assembled draft genomes were compared to the reference *L. monocytogenes* EGD-e reference chromosome using a Blast ring image generator (BRIG) [46]. The genomes were annotated to identify and label all the relevant features of a genome sequence. The genomes of our isolates were annotated using Prokka v.1.13.7 [47].

#### Data Availability

The genomes were submitted to the National Centre for Biotechnology Information (NCBI) database https://submit.ncbi.nlm.nih.gov/ (accessed on 7 May 2024) under the BioProject ID: PRJNA1108499. The metadata and accession numbers for all the isolates are attached as Supplementary Materials (Table S1).

### 2.4. MLST Analysis and Serogrouping of the Sequenced Genomic DNA

The *L. monocytogenes* genomes were subjected to MLST analysis to predict lineages, clonal complexes, and sequence types. The MLST database contains seven loci with a total of 1799 alleles. The presence and combination of these alleles enabled the prediction of *Listeria* MLST sequence types, clonal complexes, and lineages [48]. The MLST database for *L. monocytogenes* http://bigsdb.web.pasteur.fr/Listeria/Listeria.html (accessed on 23 May 2023), hosted by the Pasteur Institute in France, was used to retrieve the seven housekeeping genes, *abcZ* (ABC transporter), *bglA* (beta-glucosidase), *cat* (catalase), *dapE* (succinyldiaminopimelate desuccinylase), *dat* (D-alanine aminotransferase), *ldh* (lactate dehydrogenase), and *lhkA* (histidine kinase) for each of the *L. monocytogenes* genome. The retrieved genes were submitted to the https://bigsdb.pasteur.fr/ database (accessed on 17 May 2023), under the *L. monocytogenes* database, and the alleles and profiles database was selected from this database. The housekeeping genes were uploaded to the search by allelic profile category, and the MLST sequence types (ST), clonal complex (CC), and lineage of the *L. monocytogenes* were predicted. The prediction of serogroups was performed by submitting *L. monocytogenes* genome sequences into the database https://bigsdb.pasteur.fr/cgi-bin/bigsdb/bigsdb.pl?db=pubmlst_Listeria_seqdef (accessed on 20 May 2023).

### 2.5. Determination of Virulence Genes

The *L. monocytogenes* genome sequences were further subjected to screening for virulence gene presence using the Virulence Finder database (VFDB) through searching with ABRicate software v0.8.10, https://github.com/tseemann/abricate (accessed on 23 May 2023), a minimum cut off value of 90% was applied for both identity and coverage [49].

### 2.6. Determination of Antimicrobial Resistance Genes

Antimicrobial resistance screening involved screening the isolates for antimicrobial drug resistance, quaternary ammonium chloride (QAC), and stress resistance. The ABRicate v0.8.10 software was used with a minimum of 90% identity and coverage of 90% as cut-off values set by default settings. Antimicrobial drug-resistant genes were screened using three databases, namely, ResFinder, Complete Antibiotic Resistance Database (CARD), and NCBI, retrieved through the ABRicate v0.8.10 software. The stress tolerance genes and genes conferring resistance to disinfectants and environmental stress were retrieved from the http://bigsdb.web.pasteur.fr/Listeria/Listeria.html database (accessed on 23 May 2023), and all the isolates were screened for the retrieved genes [49]. Resistance genes identified by ABRicate v0.8.10 were validated by Blastn v.2.10.0.

### 2.7. Screening for Mobile Genetic Elements and Genomic Islands

The isolates’ genomes were screened for mobile genetic elements, specifically, plasmids, prophages, transposons, genomic islands, and insertion elements. The *L. monocytogenes* genomes were submitted to the Centre for Genomic Epidemiology database https://www.genomicepidemiology.org/ (accessed on 15 June 2023) to screen for mobile genetic elements https://cge.food.dtu.dk/services/MobileElementFinder/ (accessed on 15 June 2023) and plasmids https://cge.food.dtu.dk/services/PlasmidFinder/ (accessed on 15 June 2023). The genome of each *L. monocytogenes* isolate was submitted to the web-based database PHASTER (Prophage search tool enhanced release) database https://phaster.ca/ (accessed on 12 June 2023) used to investigate and analyze prophages. This database detected prophages into three classes, namely intact, questionable, and incomplete, based on a total score obtained by the completeness of genes needed to form an assembled virus. Only intact prophages were reported in our study.

### 2.8. Identification of the T7SS in L. monocytogenes Genome

The 24 *L. monocytogenes* genomes were screened for the presence of the T7SS essential for the secretion of surface proteins, including virulence proteins. Each of the secretion systems and the corresponding genes are outlined in Table 1. The T7SS genes used to screen each pathway found in the T7SS were outlined by Halbedel et al. [29]. The genes making up the *L. monocytogenes* T7SS were retrieved from the Gene database of the National Centre for Biotechnological Information (NCBI) https://www.ncbi.nlm.nih.gov/ (accessed on 27 September 2023). We created the T7SS database (using the genes retrieved from NCBI) using the ABRicate software v0.8.10. The *L. monocytogenes* assembled genomes and annotated genomes of our isolates were screened for the presence of the genes encoding proteins of the T7SS using the NCBI BLAST (Basic Local Alignment Search Tool).

### 2.9. Identification of Sortases

The sortases A and B were investigated by being searched on the https://bigsdb.pasteur.fr/ database (accessed on 19 October 2023). The *L. monocytogenes* database was selected from the database. The Batch sequence query was selected, the Fastq files of *L. monocytogenes* genomes were uploaded individually, and the “best match” parameter was selected. Lmo0929 (for sortase A) or Lmo2181 (for sortase B) was selected for locus search against the submitted genome sequence and finally submitted for search and identification of the gene on the sequence. The “exact match” for the locus was considered positive for sortase A or B per the respective query sequence.

## 3. Results

### 3.1. Identified Sequence Types

**Figure 1:**
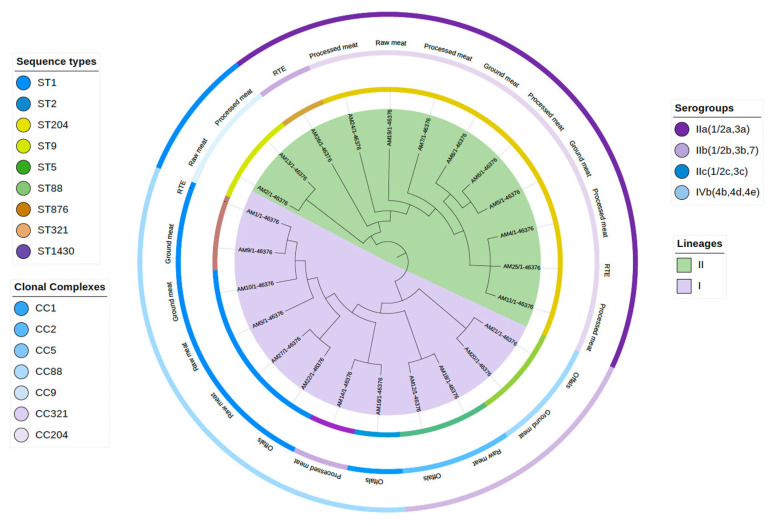
Lineages, STs, CCs, and serogroups and their association with the meat category.

MLST classified twenty-four *L. monocytogenes* isolates into nine sequence types: ST1, ST2, ST5, ST9, ST88, ST204, ST321, ST876, and ST1430; these STs were further allocated into seven clonal complexes (CC), CC1, CC2, CC5, CC9, CC88, CC204, and CC321. The isolates were classified into two evolutionary lineages: Lineage I, 46% (11/24), and Lineage II, 54% (13/24) (Table 2; Figure 1). CC204 at 42% (10/24) was the most abundant CC, while CC1 was the second most detected at 21% (5/24), and both of these CCs were found to be distributed throughout the sample categories. CC1 was represented by two STs (ST1 and ST876), and CC2 included two STs, ST2 and ST1430. The distribution of CC was not limited to the meat category but instead was unevenly distributed throughout the categories (Table 2). However, offal and raw meat represented most of the CCs (Figure 1). The isolates were grouped into four serogroups: IIa (1/2a,3a) 46% (11/24), followed by IVb (4b, 4d, and 4e) 29% (7/24), IIb (1/2b, 3b, and 7) 17% (4/24), and IIc (1/2c and 3c) 8% (2/24).

### 3.2. Identified Virulent Genes

**Figure 2:**
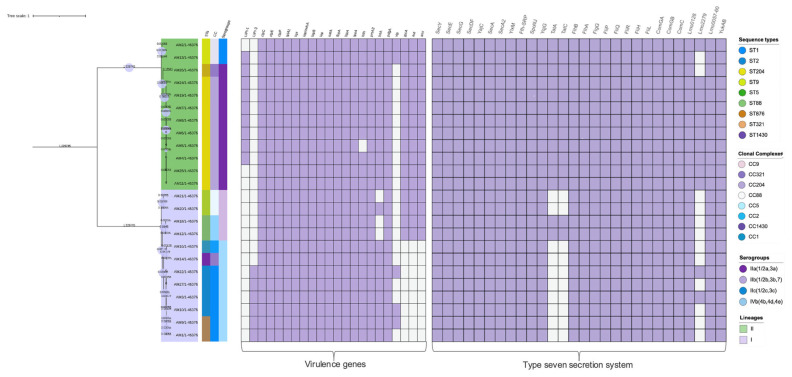
Virulence genes and the type VII secretion system detected in STs, CCs, and serogroups.

Screening for the presence of virulence genes revealed that 54% (13/24) of the isolates carried complete LIPI-1 (which consists of *hly*, *mpl*, *actA*, *plcA*, *plcB*, and *prfA* genes), and these isolates belonged to ST204, ST321, ST2, ST1430, and one of the ST9 isolates. The remaining 46% (11/24) isolates of ST5, ST1, ST88, and ST876 carried incomplete LIPI-1 that lacked the *actA* gene. The incomplete LIPI-1 was detected amongst ST204 isolates. LIPI-3 was another pathogenicity island present in a subset of isolates belonging to ST1, ST876, and ST204. Internalin genes that also play varying important roles in *Listeria* virulence were also detected in our isolates. Internalin genes *inlA*, *inlB*, and *inlC* were in all the STs, while *inlJ, inlK*, and *inlF* were only present in 58%, 54%, and 54% of the isolates, respectively. These were ST321, ST9 and ST204 isolates. All the isolates also carried the *clpC, clpE*, and *clpP* genes belonging to the clp-chaperone, *ipA1*, *hpt*, *iap/cwha*, *iapB*, *oatA*, *fbpA*, *ispA*, *ipeA*, *bsh*, *prsA2*, and *pdgA*. We also found that the virulence genes *gtcA, aut, ami, intA*, and *vip* genes were unevenly distributed amongst the STs, as shown in (Figure 2).

### 3.3. Antimicrobial Resistance

#### 3.3.1. Antimicrobial Drug Resistance Genes Identified in *L. monocytogenes*

Using the three databases (CARD, ResFinder, and NCBI), antibiotic-resistance genes were detected in all the *L. monocytogenes* isolates. The CARD database identified four resistance genes uniformly in all the isolates. The genes included *lin* conferring resistance to lincosamide, *norB* conferring resistance to fluoroquinolones, *fosX* conferring resistance to Fosfomycin, and *mprF* conferring resistance to antimicrobial peptides. The NCBI antimicrobial gene screening determined the *lin* and *fosX* genes from all the genomes. Resfinder detected the *fosX* gene conferring resistance to fosfomycin from all the isolates.

#### 3.3.2. Detected Quaternary Ammonium Chloride (QAC) Resistance Genes

Amongst the *L. monocytogenes* isolates, only 38% (9/24) were positive for the *brcABC* cassette conferring resistance against QAC-based disinfectants. These positive isolates belonged to four STs: ST204, (6/9); ST1, (1/9); ST9 (1/9); and ST321, (1/9).

#### 3.3.3. Identified Stress Resistance Genes

All the isolates were positive for stress survival islet 1 (SSI1), which comprises five genes: *lmo0444*, *lmo0445*, *lmo0446*, *lmo0447*, and *lmo0448*. No stress survival islet II from the isolates of *L. monocytogenes* was detected in the current study.

### 3.4. Identified Mobile Genetic Elements and Genomic Islands

#### 3.4.1. Plasmids Identified in *L. monocytogenes*

**Figure 3:**
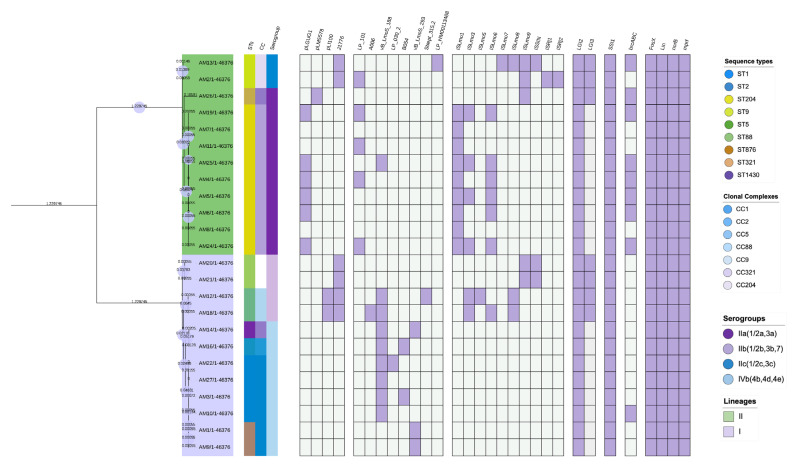
Mobile genetic elements, genomic islands, and antimicrobial resistance identified in *L. monocytogenes*.

Screening for mobile genetic elements identified four types of plasmids in 54% (13/24) of the isolates (Figure 3). The most detected plasmids were J1776 at 25% (6/24) and pLGUG1 at 25% (6/24), with J1776 detected in all ST5, ST9, and ST88 and pLGUG1 detected in six ST204. Plasmids pLI100 and pLM5578 were detected in 13% (2/13) and 7% (1/13) of positive isolates belonging to ST5 and ST321, respectively. The detected plasmids did not carry any virulence, antimicrobial resistance, or environmental adaptation genes. No transposons were detected in all the isolates.

#### 3.4.2. Prophages Identified in *L. monocytogenes*

Eight unique intact prophages, LP_101, A006, vB_LmoS_188, LP_030_2_021539, B054, Strept_315.2, vB_Lmos_293_028929, and LP_HM00113468, were identified from 71% (17/24) of the *L. monocytogenes* isolates of ST204, ST5, ST1, ST2, ST876, ST1430, and ST9 (Figure 3). Multiple prophages were detected in ST5, ST1, ST2, ST876, ST204, and ST1430 isolates. Prophage vB_LmoS_188 was the predominantly detected prophage, and it was found in all the isolates that carried multiple prophages. Diversity of the prophages in isolates belonging to the same sequence types was also observed. A total of six ST204 isolates positive for prophages were found to carry different prophages even though they belonged to the same sequence type. Isolates of ST9 also displayed a similar pattern, as these two isolates were positive for LP_101 and LP_HM00113468 prophages. A total of three isolates belonging to ST1 all carried vB_LmoS_188, and one of the isolates additionally carried LP_030_2_021539, and it was the only ST1 isolate with multiple prophages.

### 3.5. Genomic Islands and Insertion Sequences Identified in L. monocytogenes

All the isolates had *Listeria* Genomic Island 2 (LGI-2) incorporated into the genome. LGI-3 was also detected amongst some sequence types, as portrayed in Figure 3. Isolates belonging to ST5, ST9, ST88, and ST321 all had LGI-2 and LGI-3 in their genomes. A total of 38 insertion sequences were detected in 16 isolates belonging to different sequence types. A variety of 10 insertion sequences were detected: ISLmo9 (5/38), ISLmo1 (9/38), ISLmo6 (6/38), ISLmo3 (7/38), ISLmo5 (2/38), ISLmo8 (3/38), ISLmo7 (1/38), ISSN (3/38), ISRj1 (1/38), and ISRj2 (1/38). Multiple insertion sequences were identified in all the isolates. The isolates belonged to ST204, ST88, ST9, ST5, and ST321. ISLmo1 was the most detected insertion sequence and was limited to ST204, followed by ISLmo3, which was present in ST204 and ST5 isolates. ISLmo6 was also significantly represented amongst the isolates. However, it was also limited to ST204 isolates.

### 3.6. The T7SS Identified in L. monocytogenes Genomes

The T7SS in our study was identified by the characterization at the genomic level of six systems, namely, the Sec system, the Tat system, the Flagellum exporter apparatus, the Fimbrial exporter, the Holins system, and the WGX100 system. Figure 2 depicts the detection of each gene belonging to the secretion system in all the isolates. A complete T7SS was present in 42% (10/24) of our *L. monocytogenes* genomes. This is because some of the genes making up specific systems were undetected. The Sec system, the Tat system, and Holins were the only secretion systems in some of the genomes that were either absent or partially present (when some of the genes were absent). The Sec system, composed of 11 genes representing different system components, was present in all *L. monocytogenes*, but the *yqjG* gene, which forms part of the membrane integrases within the Sec system, was found to be absent in all the isolates.

### 3.7. The Identified Sortases

All the isolates contained the two known sortases in *L. monocytogenes*, SrtA and SrtB, which are essential for covalent surface protein anchoring.

## 4. Discussion

Using WGS, we could establish the population structure of our *L. monocytogenes* isolates by defining the isolates into sequence types, clonal complexes, serogroups, and evolutionary lineages. Our isolates clustered into both lineages I and II. Studies have shown that it is common for *L. monocytogenes* from food-related isolates to cluster into lineage I and lineage II evolutionary lineages [50,51,52]. Our isolates were allocated into four serogroups, with serogroup IIa being the predominant group. These serogroups include serotypes IIa (1/2a), IIb (1/2b), and IVb (4b), with 4b being the most associated with outbreaks and responsible for about 50–60% of clinical cases [53]. Serogroup IVb isolates were also detected in isolates from the 2017/2018 listeriosis outbreak in South Africa [41]. The STs and clonal complexes presented in our study have been reported from various sources such as food (ST204, ST1), food processing environment (ST9), and listeriosis cases (ST1) [35,50,54]. Although CC1 and CC2 are historically known to be predominant clonal complexes, over the years there has been an observed emergence of other CCs, such as CC5, CC6, CC9, and CC121, of which CC5 and CC9 were also detected in the current study, [55]. However, in our study, CC204 was the predominant, clonal complex, and its predominance has also been reported in studies such as Madden et al. [56] in Ireland, Melero et al. [57] in Spain, and Matle et al. [58] in South Africa. Furthermore, in South Africa, ST204 of CC204 was amongst the clonal complexes that Smith et al. [41] characterized during the 2017/2018 listeriosis outbreak, even though it was not the causative agent. Therefore, CC204 has become an important clonal complex in humans and food products.

It was also significant to characterize CC1 from our isolates, a clonal complex known to encompass clinically important isolates often associated with medical cases [59]. Despite this, CC1 is also frequently found in food products and processing environments [60,61]. CC9 and CC121 are often found in food and food processing environments [51,62]. Consequently, it was not surprising to characterize CC9 from our isolates. All the CCs that were characterized in our study, including CC204, CC1, CC9, CC2, CC5, CC321, and CC88, had been previously characterized in South Africa [38,58] from meat isolates and some in human isolates [41]. This indicates that similar CCs are circulating within the meat products in South Africa, and these CCs are likely transmitted to humans even though they have not yet resulted in a serious listeriosis outbreak since ST6 caused the largest listeriosis outbreak in South Africa.

It is important to assess the virulence potential of *L. monocytogenes* strains, as it aids in predicting the risk posed to humans [63]. The LIPI-1 identified in 46% of the isolates is significant for virulence and is frequently found in *L. monocytogenes* strains from food and food processing environments [64,65,66]. LIPI-3 is responsible for the production of Listeriolysin S, a virulence factor that plays a role in the survival of *L. monocytogenes* in polymorphonuclear neutrophils and is important for the pathogenesis of *Listeria* in humans [67], was found in a subset of the *L. monocytogenes* isolates (25%). The detection of this pathogenicity island from serogroup IVb (4b, 4d, and 4e) and (IIa 1/2a and 3a) was of serious concern, as LIPI-3 is often linked to a severe form of listeriosis in humans [68]. LIPI-3 was predominant amongst CC1 isolates, a clonal complex that is more common in human clinical cases [69]. Therefore, the transmission of our *L. monocytogenes* belonging to CC1 could result in severe and invasive listeriosis. Internalin genes identified in our study are known to code for internalins that play a vital role in the virulence mechanism of *L. monocytogenes*. For instance, the *inlA* and *inlB* genes detected in all the isolates from this study form an *inlAB* operon required for invasion during infection [70]. Identifying these internalin genes underlines the virulence potential of isolates from food products [71].

*L. monocytogenes* is generally known to be susceptible to antimicrobials acting against Gram-positives. The current study identified four antimicrobial-resistant genes, *fosX, lin, mprF*, and *norB*, conferring resistance to fosfomycin, lincosamide, antimicrobial peptide, and fluoroquinolones, respectively, in all the isolates. The presence of these genes in *L. monocytogenes* is common, and studies such as Alvarez-Molina et al. [49] in Spain, Parra-Flores et al. [72] in Chile, and Ji et al. [73] in China have reported similar results. Fosfomycin resistance is common amongst *L. monocytogenes* isolates and has been documented [64,74]. This is because *L. monocytogenes* is intrinsically resistant to these antimicrobials [75]. We did not detect the resistance genes encoding products that confer resistance to first-line antimicrobial drugs used against listeriosis. Our isolates pose a minimal threat to the current antimicrobial therapy used for listeriosis in humans. The *brcABC* cassettes were the only BC resistance genes found in 38% of our isolates. Other known BC resistance genes, such as *qacA*, *qacC*, *emrE*, *emrC*, or transposon-borne *qacH*, were not detected. The *brcABC* cassette is common amongst *L. monocytogenes* isolates from food, and Chen et al. [76] also detected the *brcABC* cassette as the only BC resistance genes from *L. monocytogenes* isolates from RTE food products. Sullivan et al. [69] identified the *brcABC* cassette only from a fraction of *L. monocytogenes* isolates, but the BC resistance genes were only found in Lineage II isolates. Like in our study, the predominant isolates positive for the *brcABC* cassette were under lineage II. Stress survival islets are some of the contributing factors for *L. monocytogenes* resistance to adverse environmental conditions [77]. All our isolates were positive for stress survival islets 1 (SSI1). This islet is required for survival under high salt concentrations, acidic pH, and biofilm formation [78,79]. This islet has also been shown to be common amongst *L. monocytogenes* from food-related isolates in studies reported by others [69,74,80]. SSI1 was demonstrated by Ryan et al. [81] to contribute to the survival of specific *L. monocytogenes* strains in suboptimal conditions. This subsequently promotes persistence in food processing environments and prolonged colonization [69,80].

Plasmids harbor genes that aid *L. monocytogenes* in environmental adaptation and resistance [82]. We identified four types of plasmids, the majority of which were identified in ST204 and ST5. These sequence types have been associated with high carriage of plasmids [83]. The absence of virulent, resistant, and environmental adaptation genes in the plasmids indicated that the plasmids do not play a role in the resistance and adaptation of *L. monocytogenes* in the current study. In South Africa, plasmids J1776, pLM5578, and pLI100 detected in our isolates were also identified by Mafuna et al. [38] from isolates obtained from meat products. Similar to our study, these authors detected J1776 in ST9 isolates and plasmid pLM5578 in ST321 isolates. Prophages play varying roles in *L. monocytogenes*, including influencing evolution, support for survival, and persistence [84,85,86]. We identified eight unique prophages from ST204, ST5, ST9, ST876, and ST1430 isolates. We also observed multiple carriages of prophages amongst ST1, ST2, ST5, ST876, ST205, and ST143 isolates. Multiple studies have shown that each *L. monocytogenes* strain can carry multiple prophages [87,88,89]. The prophages found in our isolates are documented to occur in *L. monocytogenes* from food and food-related sources [86]. The predominant vB_LmoS_188 was also found to be predominant in food isolates by Matle et al. [39]. Prophages such as vB_LmoS_188 and LP_101 have also been associated with persistence in *L. monocytogenes* [88]. Persistence as a result of prophages has also been presented in other studies [90,91,92]. Furthermore, prophages LP_HM00113468 and LP_101 from food isolates have been found to enhance *L. monocytogenes* survival and virulence [93]. Therefore, the prophages found in our study could be beneficial for the persistence and adaptation of our isolates. Genomic islands are believed to contain genetic determinants that strengthen bacteria’s survival capabilities, such as resistance to cadmium and arsenic metals [94,95]. All our isolates carried LGI-2, known to harbor genes that code for resistance against cadmium or arsenic [1]; however, this island did not carry any metal resistance genes. *Listeria* genomic island 3 (LIGI-3) was also identified in ST5, ST9, ST88, and ST321 isolates in our study. LGI-3 was identified from South African *Listeria* isolates by Mafuna et al. [96]. LGI-3 is also responsible for cadmium resistance by carrying *cadA1C* genes and is commonly associated with persistent strains of *L. monocytogenes* belonging to CC101 [73,77]. Insertion sequences identified in our study included ISLmo9 (5/38), ISLmo1 (9/38), ISLmo6 (6/38), ISLmo3 (7/38), ISLmo5 (2/38), ISLmo8 (3/38), ISLmo7 (1/38), ISSN (3/38), ISRj1 (1/38), and ISRj2 (1/38). These insertion sequences did not carry genes coding for virulence or antimicrobial resistance. A limited number of studies have found insertion sequences from *L. monocytogenes*, such as Parra-Flores et al. [72], who detected ISLmo3, ISLmo5, ISLmo7, ISLmo9, ISLmo8, and ISSN in *L. monocytogenes* isolated from RTE in Chile. The insertion sequences have also been detected in other *Listeria* species. In Egypt, Ramadan et al. [97] also found ISLmo8 and ISLmo9 from *L. innocua* isolated from milk and dairy products. ISLmo3, ISLmo4, ISLmo5, and ISLmo6 were found by Korsak et al. [98] in non-pathogenic *Listeria* species, *L. welshimeri* and *L. innocua*. Like mobile genetic elements, insertion sequences are believed to bring new genetic determinants into the bacterial genome [98]. However, there is currently limited published data on the role of insertion sequences in the virulence and environmental adaptation of *L. monocytogenes*.

For *L. monocytogenes* conversion from a saprophytic to a pathogenic lifestyle, different surface proteins are secreted via the T7SS to aid in environmental adaptation and the infection process [30]. Our study identified the complete T7SS in 42% of the *L. monocytogenes* genomes. These findings differ from the results that have been reported from the *L. monocytogenes* EGD-e genomes that have been screened for the presence of T7SS, where genes belonging to all the secretion systems were present [28,29,99]. According to our understanding, this is the first study to report the presence and characteristics of T7SS in foodborne isolates. Our primary interest was to identify the secretion systems that are crucial for the secretion of virulence proteins. According to the literature, the Sec system and the flagella exporter apparatus are the main systems that are required for virulence in *L. monocytogenes* [28,29,99].

To fully understand the virulence potential of our *L. monocytogenes* isolates, we identified the secretion system responsible for the secretion of proteins. The Sec system, the major secretion pathway for virulent proteins in Gram-positive bacteria [100], was present in all the *L. monocytogenes* isolates from the current study. Virulence proteins, phosphatidylinositol phospholipases PlcA and PlcB, Internalin C, and Hyaline (coded for by the *hly* gene), were predicted by Desvaux et al. [99] to be secreted via the Sec system. Other virulence factors that rely on the Sec system for secretion include FbpA, ActA, and Internalin B [101]. The *fbpA* and *inlB* genes were also identified in all the isolates in our study. In contrast, the *actA* gene was identified in 50% of isolates that included isolates of serogroup IIa (1/2a, 3a), IIc (1/2c, 3c), and IVb (4b, 4d, 4e), further characterized into ST204, ST9, ST2, ST1430, and ST321. It is of serious concern as these groups are often found in food, and human isolates are frequently linked to listeriosis [93]. We believe the lack of the *yqjG* gene in the Sec system in all the isolates does not hinder the secretion of proteins that are essential for virulence. This is because Halbedel et al. [29] conducted infection experiments and plaque-forming assays using *yqjG*-lacking mutants of *L. monocytogenes*, and the results proved that this gene is dispensable for virulence. Therefore, its absence does not reduce virulence.

The Sec system we characterized from our isolates contained SecA2, an important component of this system that is also significant for the secretion of virulence proteins in *L. monocytogenes* [30,102]. According to Lenz et al. [34], the secretion of the Iap (p60) protein involved in the invasion of non-phagocytic cells also occurs via the Sec system, and SecA2 is required for this protein secretion. Experimental models have shown that the mutation of the SecA2 gene reduces virulence in *L. monocytogenes* [103]. Furthermore, a study by Lenz et al. [33] showed that SecA2 is not essential for the initial stages of infection but is crucial in the later stages of disease and promotes cell-to-cell spread. Identifying secDF in the Sec system in our *L. monocytogenes* genome also amplifies the virulence potential of the isolates, as it is required for the secretion of virulence proteins [104]. SecDF was reported by Burg-Golani et al. [104] to play an essential role in the translocation of listeriolysin O (LLO), PlcA, PlcB, and ActA proteins, virulence proteins required for escape from the phagosomes and bacteria spread from one cell to the next. The *plcA*, *plcB*, *inlC*, *hly*, and *iap/cwhA* genes coding for these proteins were identified in all the *L. monocytogenes* isolates in our study.

The Flagella exporter apparatus system also plays an essential role in *L. monocytogenes* virulence [102]. In all our isolates, a complete Flagella exporter apparatus system was identified. The Flagellum export apparatus plays an essential role in the construction of the flagella and in virulence, such that the lack of the *flil* gene resulted in reduced virulence in mouse models [101]. Additionally, to provide the motility factor, the flagellum is required by *L. monocytogenes* for important surface colonization, including adhesion and biofilm formation [105]. Even though *L. monocytogenes* has six secretion systems for the secretion of proteins, experimentally, the Sec system and the Flagella are essential for virulence [101]. Even though some of these isolates are currently not of clinical significance to humans, they still pose a serious threat to humans based on the identification of virulence genes and the Sec system. The combined detection of these virulence genes with the Sec system demonstrates that *L. monocytogenes* in our study is capable of causing infections in humans should they be exposed to it and the genes be expressed.

The identification of sortases (SrtA and SrtB) from all the *L. monocytogenes* in our study was also important, as it highlighted the ability of our *L. monocytogenes* to sort and anchor LPXTG proteins on the cell surface [106]. Furthermore, the virulence of *L. monocytogenes* is extensively dependent on LPXTG proteins, making sortases A essential during infection as some virulence proteins are reportedly anchored by this sortase [107]. Virulence proteins that have been linked to sortase A for catalysis of the anchoring process include Vip protein, LapB, and internalins A, F, H, J, and K [101,108]. Having detected the virulence genes coding for these proteins in our study, these results confirm the risk of virulence posed by the *L. monocytogenes* isolated from samples in our study. In Gram-positive bacteria such as *L. monocytogenes*, the loss of sortase activity has been experimentally proven to hinder the anchoring of the LPTXG surface protein [109]. Therefore, SrtA is required for complete interaction and complete virulence of *L. monocytogenes* [108].

## 5. Conclusions

In conclusion, our study provided data on the population structure and molecular characteristics of *L. monocytogenes* in beef and beef-based products sold in retail outlets in two provinces in South Africa. This study showed that ST204 was more frequent amongst the isolates, and clinically important STs such as ST1 and ST2 are circulating within the beef products. *L. monocytogenes* are capable of virulence as they possess virulent factors required for pathogenesis. Enhanced virulence was observed in CC1 isolates, a clonal complex of clinical importance in humans. Our isolates did not carry genes that can resist antimicrobial drugs used as a first-line defense against listeriosis. However, these isolates did carry genes that enable environmental resistance. A characteristic that could promote persistence in the food environment. The T7SS and sortase A are common amongst food isolates, and therefore, our isolates are potentially able to translocate and anchor proteins into the extracellular space.

## Figures and Tables

**Table 1:** Genes used for screening of the T7SS in *L. monocytogenes*.

The Secretion System	The Component	Genes
The Sec system	SecY	*lmo2612*
	SecE	*lmo0245*
	SecG	*lmo2451*
	SecDF	*lmo152*
	YajC	*lmo1529*
	SecA	*lmo2510*
	SecA2	*lmo0583*
	FtsY	*lmo1803*
	YlxM	*lmo1802*
	Ffh	*lmo1801*
	SpolllJ	*lmo2854*
	YqjG	*lmo1379*
The Tat system	TatA	*lmo0362*
	TatC	*lmo0361*
Flagella Exporter	FlhBA	*lmo0679-0680*
	FliO	*lmo0682*
	FliPQR	*lmo0676-0678*
	FliH	*lmo0715*
	Flil	*lmo0716*
Fimbrial Exporter	ComGA	*lmo1347*
	ComGB	*lmo1346*
	ComC	*lmo1550*
The Holins	Lmo0128	*lmo0128*
	Lmo2279	*lmo2279*
	Lmo0057-60	*lmo0057-60*
	YukAB	*lmo0061*

**Table 2:** Distribution of *L. monocytogenes* clonal complexes in meat categories.

Meat Category	Lineage I				Lineage II			
	CC1	CC2	CC5	CC88	CC9	CC204	CC321	Grand Total
Ground meat	2			1		2		5
Offal	1	1	1	1				4
Processed meat		1			1	5		7
Raw meat	1		1		1	2		5
RTE	1					1	1	3
Grand total	5	2	2	2	2	10	1	24

## Data Availability

Data are contained within the article and Supplementary Materials.

## Supplementary Materials

The following supporting information can be downloaded at: https://www.mdpi.com/article/10.3390/microorganisms12061166/s1, Table S1: Genome sequences with metadata and accession numbers.
