# Supplementary material for: Virulence Potential and Antimicrobial Resistance of Listeria monocytogenes Isolates Obtained from Beef and Beef-Based Products Deciphered Using Whole-Genome Sequencing
Source: Microorganisms. 2024 Jun 8;12(6):1166. doi: 10.3390/microorganisms12061166 (PMC11205329; doi:10.3390/microorganisms12061166)
Supplement: Supplementary file 1 [file microorganisms-12-01166-s001.zip › microorganisms-3002753-supplementary.pdf]

Table S1; Genome sequences with metadata and accession numbers

Place of isolation	*Listeria* species	ST	CC	Lineages	Serogroup	Accession number	Type of sequences	Contig count	N50	GC Content (%)	Date of collection
North West	*L. monocytogenes*	204	204	II	IIa(1/2a,3a)	SAMN41244492	Raw reads	65	391095	37,92	21/10/2024
North West	*L. monocytogenes*	5	5	I	IIb(1/2b,3b,7)	SAMN41244487	Raw reads	67	369508	37,88	21/10/2024
North West	*L. monocytogenes*	321	321	II	IIa(1/2a,3a)	SAMN41244494	Raw reads	36	545408	37,84	21/10/2024
North West	*L. monocytogenes*	204	204	II	IIa(1/2a,3a)	SAMN41244493	Raw reads	30	391095	37,92	21/10/2024
North West	*L. monocytogenes*	88	88	I	IIb(1/2b,3b,7)	SAMN41244489	Raw reads	68	603789	37,9	21/10/2024
North West	*L. monocytogenes*	1	1	I	IVb(4b,4d,4e)	SAMN41244491	Raw reads	73	267072	37,85	21/10/2024
North West	*L. monocytogenes*	204	204	II	IIa(1/2a,3a)	SAMN41244488	Raw reads	29	391095	37,94	21/10/2024
North West	*L. monocytogenes*	2	2	I	IVb(4b,4d,4e)	SAMN41244486	Raw reads	46	321339	37,84	21/10/2024

Mpumalanga	*L. monocytogenes*	204	204	II	IIa(1/2a,3a)	SAMN41244475	Raw reads	246	391095	38,77	21/10/2024
Mpumalanga	*L. monocytogenes*	204	204	II	IIa(1/2a,3a)	SAMN41244476	Raw reads	57	423134	38,03	21/10/2024
Mpumalanga	*L. monocytogenes*	204	204	II	IIa(1/2a,3a)	SAMN41244477	Raw reads	136	260109	38,07	21/10/2024
Mpumalanga	*L. monocytogenes*	204	204	II	IIa(1/2a,3a)	SAMN41244495	Raw reads	130	267072	38,16	21/10/2024
Mpumalanga	*L. monocytogenes*	5	5	I	IIb(1/2b,3b,7)	SAMN41244483	Raw reads	48	493484	37,85	21/10/2024
Mpumalanga	*L. monocytogenes*	876	1	I	IVb(4b,4d,4e)	SAMN41244472	Raw reads	112	557757	37,94	21/10/2024
Mpumalanga	*L. monocytogenes*	1	1	I	IVb(4b,4d,4e)	SAMN41244474	Raw reads	362	522325	39	21/10/2024
Mpumalanga	*L. monocytogenes*	876	1	I	IVb(4b,4d,4e)	SAMN41244480	Raw reads	70	558125	37,81	21/10/2024
Mpumalanga	*L. monocytogenes*	204	204	II	IIa(1/2a,3a)	SAMN41244479	Raw reads	44	397232	37,94	21/10/2024
Mpumalanga	*L. monocytogenes*	1	1	I	IVb(4b,4d,4e)	SAMN41244481	Raw reads	86	480542	37,69	21/10/2024
Mpumalanga	*L. monocytogenes*	1430	2	I	IVb(4b,4d,4e)	SAMN41244485	Raw reads	52	321334	37,88	21/10/2024

Mpumalanga	*L. monocytogenes*	9	9	II	IIc(1/2c,3c)	SAMN41244484	Raw reads	36	476310	37,88	21/10/2024
Mpumalanga	*L. monocytogenes*	88	88	I	IIb(1/2b,3b,7)	SAMN41244490	Raw reads	60	603766	37,89	21/10/2024
Mpumalanga	*L. monocytogenes*	9	9	II	IIc(1/2c,3c)	SAMN41244473	Raw reads	2792	59058	39,89	21/10/2024
